# The Particularities of Pharmaceutical Care in Improving Public Health Service during the COVID-19 Pandemic

**DOI:** 10.3390/ijerph18189776

**Published:** 2021-09-16

**Authors:** Steliana Ghibu, Anca Maria Juncan, Luca Liviu Rus, Adina Frum, Carmen Maximiliana Dobrea, Adriana Aurelia Chiş, Felicia Gabriela Gligor, Claudiu Morgovan

**Affiliations:** 1 Department of Pharmacology, Physiology and Pathophysiology, Faculty of Pharmacy, “Iuliu Haţieganu” University of Medicine and Pharmacy, 6A Louis Pasteur Street, 400349 Cluj-Napoca, Romania; stelianaghibu@yahoo.com; 2 Preclinical Department, Faculty of Medicine, “Lucian Blaga” University of Sibiu, 2A Lucian Blaga St., 550169 Sibiu, Romania; ancamaria.juncan@ulbsibiu.ro (A.M.J.); liviu.rus@ulbsibiu.ro (L.L.R.); adina.frum@ulbsibiu.ro (A.F.); carmen.dobrea@ulbsibiu.ro (C.M.D.); felicia.gligor@ulbsibiu.ro (F.G.G.); claudiu.morgovan@ulbsibiu.ro (C.M.)

**Keywords:** pharmaceutical care, pharmaceutical services, pharmaceutical practice, public health, COVID-19, pharmacist, pharmacy, healthcare

## Abstract

Nowadays, humanity is confronted with one of the most difficult challenges. Severe Acute Respiratory Syndrome Coronavirus 2 (SARS-CoV-2) was identified for the first time in Hubei, China in December 2019 and produced the COVID-19 pandemic, a devastating disease that led to many complications and deaths. The authorities and the global healthcare system have been alerted regarding the prevention and treatment of this pathology. Even though worldwide quarantine was declared, health care professionals, including pharmacists, have been at the frontline in this war. Since the beginning of the pandemic, the authorities relied on the involvement of the community, hospital, or clinical pharmacists in offering support to the entire population. Also, the authorities implemented measures for emergency authorization of the vaccines, or the drugs used in COVID-19 treatment. In order to facilitate the population’s access to healthcare services, the authorities have established regulations regarding, the extension of prescriptions by pharmacists, working hours, prevention of shortages and price-increase, drive-thru services, etc. However, several countries have taken financial measures to support the pharmacies’ activity. At the same time, pharmaceutical associations elaborated guidelines for the protection of pharmacists and patients alike. Additionally, the pharmacies have come to support the health system and patients by adapting pharmaceutical care to the new needs like preparation and supply of disinfectants, patient care, information, and counseling, especially to COVID-19 patients, as well as the implementation of home drugs-delivery systems. The important roles played by pharmacists were to perform COVID-19 tests and further vaccines, as well as to combat the abundance of misinformation and fake news. The clinical and hospital pharmacy services have also been adapted. Strengthening the role of the pharmacist in the medical team was important for the purpose of providing correct and complete information regarding drugs used in the COVID-19 pathology. In all these activities, pharmacists needed creativity and professionalism, but also the support of pharmacy owners and managers. With this crisis, pharmaceutical care has entered a new phase, demonstrating the ability of pharmacists to be competent and accessible providers of public health. Based on this information, we conducted a narrative review whose purpose was to identify the impact of the authorities’ decisions on pharmaceutical practice, the involvement of professional associations, and the responsibilities of the pharmacy owners and management. On the other hand, we performed a global assessment on the pharmaceutical care services provided by community pharmacists as well as by clinical or hospital pharmacists during the COVID-19 pandemic.

## 1. Introduction

The end of the year 2019 had a dramatic impact on all humanity. At the same period, on 26 December 2019, in PlosOne journal, Watson et al. identified 43 possible roles of pharmacists in critical situations [1]. All these findings showed the importance of pharmacy as a medical profession, in order to support or to complete the practice of physicians [1]. A few days later, forced by the circumstances, pharmacists had to gradually demonstrate, their capacity to apply these skills. The first cases of pneumonia caused in the world by an unknown agent were announced (on 31 December 2019) [2]. Further, the researchers established that Severe Acute Respiratory Syndrome Coronavirus 2 (SARS-CoV-2), a new type of Coronavirus (Coronaviridae Family), produced the infection and was also responsible for a more complex, COVID-19 disease [3]. The rapid spread of this disease and the occurrence of severe cases that triggered major organ damage, including death, led authorities around the world to declare lockdown successively, and on 11 March 2020, WHO declared the COVID-19 pandemic [4,5,6]. This pathology affected the whole society, at all levels (medical, social, economic, psychological, etc.), disrupting the activity of the entire population. During the pandemic period, many researchers focused on different COVID-19 issues.

Pharmaceutical care represents a concept introduced after 1990. According to this, the pharmacists’ activity focuses on patients and aims to provide adequate therapies that lead to safe therapeutic results, as well as to improve the quality of life [7]. Thus, the traditional activity of preparation and development of drugs has been gradually replaced by pharmaceutical services that mostly focus on the patients’ needs and the particularities of their pathologies [8,9]. But, with the onset of this pandemic, the activities of all professional categories, including the pharmaceutical activities, have been disturbed and this crisis deeply and continuously marked the practice of pharmacists [10]. Pharmacists are considered the most accessible professionals from the health sector and they support the medical practice [11]. Bragazzi et al. summarized the multiple roles of community or hospital pharmacists, such as experts in drugs and medicines, healthcare providers and stakeholders, educators, counsellors, mentors, managers, leaders, business developers, researchers, etc. [12]. 

Pharmacists are a valuable human resource, being at the center of their communities. Based on their professional education and their scientific activity, they can provide correct and complete medical information to the population [13,14]. Since the beginning of the pandemic, pharmacists remained in the first line in order to help their patients and to prevent and combat the infection’s effects. During this period the pharmaceutical practice diversified, some authors considering that it passed into a “new era” [12,15]. 

The aim of this study was to identify the impact of the authorities’ decisions on pharmaceutical practice, the involvement of the professional associations, and the responsibilities of pharmacy owners and management. On the other hand, we performed a global assessment on the pharmaceutical care services provided by the community, clinical, or hospital pharmacists. 

To accomplish this study, we conducted a narrative review of the main articles identified in Pubmed, by using specific keywords: “COVID-19” or “Coronavirus” and “pharmaceutical care”, “pharmaceutical services”, “pharmacy(ies)” or “pharmacist”. Subsequently, the information was structured in some main subheadings, starting from the decision-makers to the final link of the pharmaceutical care, the pharmacist.

## 2. The Impact of the Authorities’ Decisions on Pharmaceutical Care

### 2.1. General Recommendations of the Authorities

Health is a fundamental right of every human being and it represents the absence of disease or infirmity and a state of well-being. All healthcare professionals have provided proactive services to prevent, cure or rehabilitate people’s health [16,17,18]. WHO recognized the important role of community pharmacies in offering support for people with COVID-19 symptoms. Thus, it was recommended that pharmacists should advise people about the necessity of hygiene and social distancing. Additionally, pharmacists had to combat the fake news or misinformation, start home-delivery services, and extend their work schedule [19]. Since the beginning of the crisis, the Centers for Disease Control and Prevention (CDC) in the USA drafted a guide for pharmacies in order to protect both staff and patients [20]. Simultaneously, the European Center for Disease Prevention and Control also drafted guidelines for pharmacies [21]. Subsequently, other institutions made recommendations for pharmacies. On 24 February 2020, community pharmacies in Italy received some recommendations regarding their practice during the regional critical period. On the other hand, pharmacies had to stay open, but they had to respect the social distancing, and the staff had to use personal protective equipment (PPE) [22]. In some countries (e.g., Croatia), the limited PPE supply had determined the authorities to force the pharmacies’ staff to wear face masks only after 13 July 2020 [23]. 

Since 22 March 2020, the National Health Services (NHS) from the United Kingdom (UK) developed some standard operating procedures and guides for community pharmacies that included general principles of service during the COVID-19 pandemic. These refer to: (a) staff (risks, testing, approach in case of symptoms or infections, preventive measures, etc.), (b) pharmaceutical care services, (c) preparation activity, (d) supporting, informing and managing the patients, including COVID-19 patients, etc., (e) proper ventilation and disinfection of pharmacies, (f) telephone counseling, g) home delivery services [24,25].

In Spain, procedures regarding pharmaceutical care and pharmacy staff protection were elaborated by the health authorities [26]. The Croatian Institute of Public Health and the Croatian Chamber of Pharmacists recommended pharmacies to split the shifts. According to this recommendation, many pharmacists from Croatia worked 7 h/day for 7 days, and then 7 days they had to stay at home [23].

Although in many countries pharmaceutical authorities had a rapid reaction to the pandemic and an unexpected reaction was reported in others. Thus, Atif et al. noticed that in Pakistan, healthcare authorities did not have any reaction to support or to guide the pharmaceutical services needed to attenuate the COVID crisis. This lack of reaction could be attributed to a low consideration for the activity of pharmacies. The authors’ opinion was that pharmaceutical entities were not healthcare settings and they were rather compared by the authorities to ordinary commercial locations [27]. Another similar situation was encountered in Lebanon and Zimbabwe, where the pharmacists were not engaged in the health emergency plans [28,29]. 

### 2.2. Emergency Use Authorization

The emergency use authorization (EUA) allows the authorization and use of drugs, including vaccines, during public health emergencies. This authorization was based on the relevant statutory criteria and took into account all scientific evidence about the drug [30]. Since the beginning of the pandemic, the Food and Drug Administration (FDA) has had a major role in the authorization of different treatments related to the COVID-19 disease. So, we can refer to the EUA issued on 28 March 2020, for hydroxychloroquine sulphate and chloroquine phosphate for the COVID-19 treatment [31] (revoked later on 15 June 2020 [32]) or to the acceleration of tests for new drugs (including vaccines). Making a hasty or uncoordinated decision to authorize a new treatment can cause distrust in scientists, health officials, and regulators [33] beyond drug inefficiency or the risk of adverse reactions (ADRs), including serious ones. 

### 2.3. Regulations Regarding Drugs Prescriptions 

The community pharmacies in some countries (France, Ireland, Italy, Malta, Portugal, or Serbia) were legally allowed to renew prescriptions for chronic diseases. In Austria, Brazil, Canada, France, etc. even hypnotics, anxiolytics, narcotics, and opiate substitution treatments were allowed [34,35]. However, the pandemic reduced access to medication for opioid addiction (buprenorphine, methadone, etc.). Thus, pharmacists are qualified and capable to ensure the continuity of treatment, and they could be used to facilitate access to drugs [36].

As in many countries, in Italy, paper-based prescriptions were replaced with e-prescriptions [22]. In Romania, the pharmacies were able to dispense medicines based on prescriptions transmitted by electronic means, excepting prescriptions for narcotic and psychotropic drugs [37]. In Australia, the authorities facilitated the uploading and sending of electronic prescriptions [14] and pharmacists were empowered until 30 June 2020, to supply subsidized drugs without a prescription (for up to one month) if the drugs were previously prescribed [38].

### 2.4. Regulations of Working Hours

Although the authorities in every country established lockdown, the working hours of pharmacies were not restricted. During the state of emergency, pharmacies were advised to stay open according to their normal schedule or even to extend their work hours. For example, in the UK, according to NHS guidelines, pharmacies can be closed for a maximum of 2.5 h per day [24]. However, in Croatia, the Crisis headquarter reduced the working hours of pharmacies to a maximum of 7 h/day (from 8 AM to 5 PM), apart from on-duty pharmacies, while in Serbia, only the schedule of pharmacies located in malls were affected [23]. A study performed in Romania showed that pharmacies most frequently adjusted their working hours depending on the requirements and conditions generated by the state of emergency [37].

### 2.5. Legislative Regulations for Preventing Shortages or Drug Price Increases

An increasing shortage of drugs and PPE was observed in the first stages of the pandemic because of the disruption in manufacturing, high demand, shipment delay, and shortage of ingredients [39]. The COVID-19 pandemic excessively increased the demand for PPE (masks, gloves, etc.), sanitizers, dietary supplements (vitamin C, vitamin D, zinc, and melatonin, etc.), and drugs, many of which were used off-label (e.g., chloroquine, hydroxychloroquine, remdesivir, lopinavir, azithromycin, glucocorticoids, etc.) [28]. On the other hand, dietary supplements overuse might have ADRs [40]. Thus, pharmacists once again played an important role in limiting irrational consumption of drugs. 

The excessive media coverage of chloroquine and hydroxychloroquine led to an increased demand for these drugs [23]. In April 2020, the American Society for Health-System Pharmacists listed 210 new active shortages [41]. The pharmacists from Denmark reported an insufficient quantity of propofol in intensive-therapy units [42]. The causes for drugs shortages in Rwanda were the reduction of drug imports and unjustified buying practices generated by the panic [43]. 

Under these circumstances, the pharmacies were confronted with many shortages, some of them temporary, others long-term. Therefore, pharmacists had to manage the stock of these drugs very carefully in order to ensure especially the treatment of patients with rheumatoid arthritis, systemic lupus erythematosus, and other autoimmune disorders [15,39,44]. 

The authorities from many countries took different decisions to reduce drug shortages, such as:

(i) the substitution of drugs with similar ones or dose adjustments were allowed for pharmacists from different countries (e.g., Australia, Belgium, Croatia, Germany, the Netherlands, Portugal, UK, etc.);
(ii) in Denmark, health authorities demanded pharmacies to report the stock of critical medicine daily;
(iii) in Croatia, France, Malta, Portugal, and Spain, community pharmacies could dispense some drugs used in hospitals, and some studies showed that the patients were satisfied with this service provided by the community pharmacy [35,38,42];
(iv) limiting the drugs dispensed from the pharmacy was another legislative measure to prevent shortages. In Australia, the number of medical specialists that could initiate the treatment with hydroxychloroquine was restricted, or the quantity released off-label was of maximum one box/month/patient or one prescription/month/patient [14]. The Estonian Health Ministry limited the quantity of paracetamol to 2 boxes/patient [42]. The Saudi-Arabian Health Ministry limited the quantity of dietary supplements that boost the immune system to only one pack per customer to prevent the emergence of a black market for such items [45];
(v) in Rwanda, the Ministry of Trade and Industry applied fines to pharmaceutical companies for increasing drug prices. In order to reduce price speculation and drug shortages, the authorities sent pharmacies a list of drugs in high demand, including their prices [43]. On the other hand, the healthcare insurance company from Croatia allowed pharmacies to dispense drugs with higher prices than the regulated ones, the difference being supported by health insurance. Also, these measures were enforced to solve drug shortages and limit the return of patients in pharmacies [35].

### 2.6. Oxygen Therapy

Severely affected COVID-19 patients required oxygen therapy [46,47]. In this context, an increase in the need for medical oxygen was noticed. Authorities from various countries have also involved pharmacists in distributing the oxygen needed for home care to support this excessive demand. For example, in Italy, a decree allowed pharmacists to provide oxygen for home-care therapy [48,49]. Likewise, in Belgium, the pharmacists could order the oxygen (only prescription-based) from suppliers for ambulatory care (oxygen concentrator, gaseous and liquid oxygen) [50]. Taking into account that the oxygen is very inflammable and its at-home administration involved many risks [51], pharmacists advised patients regarding the correct manipulation. 

### 2.7. Drive-Thru Pharmacy

The drive-thru pharmacy concept has been in use for many years in the United States of America (USA), and, in the last year, other countries (Australia, Croatia, Denmark, Jordan, Malaysia, Qatar, Taiwan, UK, USA, etc.) have launched these services. Namely, people can pick up the drugs from outside the pharmacy, directly from their car [35,52]. Some of the noticed advantages are the improvement in pharmaceutical care services for seniors or parents with small children, time-saving, parking convenience, etc. Although this service has some notable disadvantages (processing delays, reduced efficiency, dispensing errors), during the pandemic period, other advantages can be attributed to this system (e.g., ensuring and improving the safety of pharmacy staff and patients) [52].

### 2.8. “Mask 19” Code

“Mask 19” is a code used in pharmacies from different countries (Austria, Belgium, France, Greece, Italy, the Netherlands, Norway, Portugal, Spain, etc.) by victims of domestic violence. During the lockdown period, the victims of domestic violence were forced to spend substantial amounts of time together with their abusers. In this context, the incidence of domestic abuse was greater and, for these victims, the pharmacy represented “a community hub and a safe haven“ offering the best way to report their abusers. In a private discussion with the pharmacist, the victims pronounced “Mask 19” a simple code that does not attract much attention even if they are not alone. For these reasons, since the beginning of the pandemic, the authorities established that pharmacies represent a safe and trusted environment for domestic violence victims [35,53,54,55,56]. Furthermore, Her Majesty’s Government from the UK elaborated guidance for pharmacies in order to offer support for victims of domestic abuse to access immediate help from the police or other authorities [57,58].

### 2.9. Financial Aspects Regarding the Pharmaceutical Services

In most countries, pharmaceutical care services were not remunerated. However, some of them allowed pharmacies to provide paid services for:

(i) rapid antigen tests performed in pharmacies which were reported to the Ministry of Health (Cyprus) [35];
(ii) specific COVID-19 pharmaceutical services provided by pharmacies (New Zealand, Scotland, Germany) [59,60];
(iii) vaccination of patients (Australia) [39].

In order to facilitate the financing of pharmacies, the Polish authorities decreased the time of fund reimbursement to pharmacies and allowed pharmacists to prescribe subsidized drugs for themselves and their closest relatives [48].

## 3. The Involvement of the Professional Associations

Together with physicians and nurses, pharmacists were one of the professional categories most exposed to the risk of SARS-CoV-2 infection since the pandemic onset. In addition, they were among the most important players against the virus. Worldwide, pharmacist organizations quickly took action in order to protect their members and patients. Since the beginning of this pandemic, the Chinese Pharmaceutical Association elaborated some guidelines for preventing and controlling the SARS-CoV-2 infection. Thus, the pharmacists from hospital or community pharmacies and all staff from Chinese pharmaceutical warehouses had a working tool against COVID-19 at their disposal. Furthermore, Chinese pharmacists have been trained to use PPE, sterilize work environments, or ameliorate mental stress [61].

Based on these recommendations, after only six days from the official announcement when WHO declared COVID-19 a Public Health Emergency of International Concern (PHEIC), the International Pharmaceutical Federation (FIP) published the first guide for pharmacists around the world. Addressed to more than 4 million pharmacists, “Coronavirus 2019-nCoV Outbreak” included information and interim guidelines for pharmacists and the pharmacy workforce. The document was updated on 19 March 2020. The guidelines referred to (a) SARS-CoV-2 transmission and incubation, (b) diagnostic tests, (c) preventive measures (sanitization, disinfection, mask-wearing, travel advice, etc.), (d) treatment (specific drug information, potential vaccines, patient counseling, streamlining the supply of medicines, etc.) and (e) rules to ensure safety and continuous pharmaceutical services [62,63]. 

**Figure 1:**
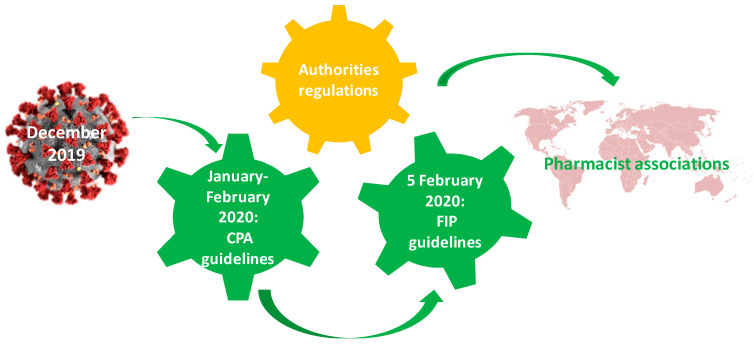
The mechanism of elaborating the pharmaceutical guidelines on COVID. CPA—Chinese Pharmaceutical Association; FIP—International Pharmaceutical Federation.

Based on FIP guidelines, the professional authorities from every country (e.g., the American Medical Association—AMA, the American Society of Hospital Pharmacists—ASHP, the American Society of Health-System Pharmacists—ASHSP, Pharmaceutical Group of the European Union—PGEU, the Pharmaceutical Society of Australia—PSA etc.) had been formulating specific recommendations for their members (Figure 1) [13,29,64]. 

Moreover, in the USA, the legislation in different states was amended, so that it allowed the practice of different categories of pharmacists (inactive, authorized in another state or with an expired license, etc.). On the other hand, in other states, the expired license date was extended or the conditions for licensing of pharmacists were relaxed [31]. 

Several ways in which the professional associations got involved in the pharmaceutical care support are mentioned below:

(i) PGEU elaborated some recommendations in order to protect the pharmacists and the patients, to ensure the optimal stocks, to identify and to report the suspected COVID-19 cases by pharmacists, to produce the hand disinfectants, to advise and inform the patients regarding different pathologies or treatment including COVID-19 ones, to deliver drugs at patients’ home. [64]. These recommendations were sent to pharmacists by means of the national professional organizations.
(ii) The Croatian Pharmacy Chamber made specific recommendations for the pharmacists regarding safety measures, restrictions concerning the dispensation of specific drugs or PPE [23].
(iii) In Serbia, the FIP guidelines were translated in the Serbian language by the Centre for Pharmacy and Biochemical Practice Development—University of Belgrade and the Pharmaceutical Chamber of Serbia [23].
(iv) The official journal of the Royal Pharmaceutical Society from the UK (Pharmaceutical Journal) presented a material elaborated for pharmacies by CDC in order to help them manage the cold and flu during the COVID-19 pandemic [65].
(v) The pharmacists’ association from Spain (Consejo General de Colegios Farmacéuticos) offered people correct information about COVID-19 vaccines, fought fake news and misinformation [66].
(vi) The French Society for Oncological Pharmacy elaborated some recommendations in order to improve the pharmaceutical care for cancer patients during the pandemic. In addition to the general rules, during the pandemic, this guide contained some specific rules, such as (a) the pharmacy staff moves to other departments only when needed and the monitoring and the information will be collected remotely by using the telephone, online, by means of electronic files, etc., (b) the cytostatic production units have to be bio-cleaned under controlled atmosphere, (c) the gloves should be frequently changed; (d) injectable forms should be replaced with oral forms, if possible, (e) relocation of students in practice to the sections with deficient staff, (f) pharmacists have to monitor the stocks of glucocorticoids, anti-inflammatories or kinase inhibitors (drugs used experimentally in COVID-19), etc. [67].
(vii) The American Society of Hospital Pharmacists gave patients 60-day free access to its own database with COVID-19 pathology and treatment [13].
(viii) In Australia, authorities, together with the Pharmacy Guild of Australia and PSA, allowed the substitution of drugs with similar ones or dose adjustments by pharmacists when a prescribed drug was unavailable. Also, PSA supported the pharmacists in educating the population to encourage rational drug purchasing (even by drafting posters) [14].
(ix) In Lebanon, the Order of Pharmacists elaborated some minimal guidance for community pharmacists [29].

## 4. Responsibilities of the Owners and Management of Pharmacies

One of the main responsibilities of pharmacy owners or staff management consisted in protecting the employees from the SARS-CoV-2 infection. Some noticeable measures taken by the owner and/or manager were: (a) provision of PPE and disinfection products, (b) elaborating workplace safety protocols, (c) monitoring the employees’ health status, (d) training the employees, (e) distancing and protecting personnel (e.g., split shifts, face shields, plexiglass or acrylic glass shields, dispensation through the window, quarantine room, extensive cleaning, and disinfection of surfaces), (f) restricting patient numbers for concomitant access [31,42,45,68]. The services provided to patients were also limited in pharmacies, (e.g., cancellation of face-to-face meetings, interruption of blood pressure, glycaemia, and cholesterol measurements, provision of home testing kits, provision of a home treatment plan, cancellation of spirometric testing of asthmatic patients, etc. [42]. Another aspect of managerial activity was the rationalization of the drugs or medicines in order to avoid drug or PPE shortages (e.g., Serbia, Croatia) [23]. 

## 5. COVID-19 Influences on Pharmacists’ Health Status

As front-line workers, pharmacists were exposed to the risk of SARS-CoV-2 infection, whether as direct contact with the patient in the dispensary space, with suppliers for restocking, with other people during their travel to work by public transport, or when dispensing paper-based prescriptions [28]. 

Ashiru-Oredope et al. showed in a study that the majority of pharmacist respondents were worried or very worried about the impact of COVID-19 on their personal life or professional activity [69]. 

A lot of problems were identified in the European countries: staff relocation to new workspaces or activities, cancellation of holidays (e.g., England), restriction of the activity in the community pharmacies by the authorities (e.g., Croatia), lack of access to staff testing, the impossibility of implementing staff distancing, limited access to electronic documents in case of work from home, lack of clinical knowledge about the use of drugs in COVID-19. Additionally, low quality and quantity of PPE and disinfectants and the lack of knowledge about the correct usage of PPE was reported in Romania and Turkey. All these aspects can affect health status [37,42]. 

Other studies show that fear of workplace infection was another negative influence of the pandemic, both for pharmacists and their family members. Fatigue, burnout, stress, fear, anxiety, trauma, affected productivity, or worsening in the communication of pharmacists with their colleagues were the main risks for pharmacists during this period [23,31,42,69,70].

In this context, the health-related quality of life of community pharmacists from Romania and Bulgaria during the pandemic was studied. The results showed lower levels of quality of life for the Bulgarian group referring to sleeping, mental function, depression, distress, etc. [10]. Another negative aspect noticed by Novak et al. [23] was the sentiment of dissatisfaction concerning the public perception of the Serbian pharmacists’ role. In a study conducted in April 2020, the Canadian Pharmacists Association (CPhA) showed that, since the onset of the COVID-19 pandemic, more than 70% of the pharmacists complained of increased harassment from patients, verbal or other forms of abuse [71]. Ebeldini et al. showed that pharmacists from the USA, UK, or France faced anti-Asian racism and physical abuse which affected their wellbeing status [72]. 

In order to help pharmacists maintain good mental health, professional associations (e.g., CPhA) broadcasted information through various media [72,73]. Moreover, the authorities in Wyoming USA created an online group in order to support the pharmacists and other healthcare categories against mental imbalances caused by the pandemic [31].

## 6. Pharmaceutical Care in Community Pharmacies

For the success against the COVID-19 pandemic, the authorities included the community pharmacists in the public health team [74]. A study conducted in Saudi Arabia confirmed that all questioned pharmacists were prepared for this role [75]. Every decision of each pharmacist has to reduce the number of patient visits to the pharmacy or doctors and to control the risk of infection [68]. 

Visacri et al. identified the main roles of pharmacists during the pandemic: (a) prevention and infection control, (b) provision, storage and supply of PPE and drugs, (c) patient care, and support for healthcare professionals [76]. 

In addition, Bragazzi et al. highlighted the important role of pharmacists in screening, triage, detection, reporting of potential COVID-19 cases, active surveillance, and early alerts to drug shortages, telepharmacy services, combating fake news and misinformation regarding the COVID-19 medication, etc. [12]. Elbedini et al. noticed the role of pharmacists in physical evaluation, blood pressure measurement, fever monitoring, and COVID-19 testing [77]. 

One of the most advanced health systems was in Saudi Arabia. During this period, the pharmacist’s role was very well established in this country. The services identified by Ahmad et al. and provided by community pharmacists were: public information (including the elaboration of different materials), patient counseling (especially online counseling of lockdown patients), treatment delivery and follow-up, drug misuse prevention, chronic disease management, reporting people suspected of COVID-19 [11].

Following these aspects, a study conducted in the Netherlands, showed that the main influences in the community pharmacies activity were: (a) hygienic rules, (b) logistic changes (working in shifts, plastic screens, etc.), (c) limited visits in the pharmacy (online prescriptions, waiting lines, special pick-up counters, self-service lockers, home delivery, etc.), (d) alternative ways for medication review (by phone or by videoconference, etc.), (e) providing the information (e.g., flyers, web pages, video animation, etc.); (f) collaboration with doctors (most frequently by phone) [78].

### 6.1. Production of the Antiseptic Agents

At the beginning of the pandemic, the stocks of disinfectants were limited. For this reason, pharmacists had begun to produce various disinfectants, especially 70% alcohol-based products, following WHO recommendations. This activity took place in the community and hospital pharmacies in many countries (e.g., Austria, the Czech Republic, Denmark, France, Finland, Germany, Italy, the Netherlands, Poland, Sweden, UK, etc.) [37,48]. On the other hand, disinfectants were produced in the faculties of pharmacy laboratories (e.g., Brazil, Romania, etc.), from where they were to be used for their own operations, for various medical units, or other institutions [79,80].

### 6.2. Patient’ Triage, Information and Counseling

Mallhi et al. highlighted that community pharmacists are “the most accessible and underused” health system resource [81] and an important source of valid information on pathology and disease control [72]. During the pandemic, pharmacists were actively involved in patients’ triage, information, and counseling. Carried out either face-to-face, by telephone or online, the triage consisted of screening COVID-19 symptoms and often required redirecting the patient to other medical service providers. Direct contact required adequate PPE, often unavailable in pharmacies, and this was an important stress factor and increased infection risk [14,72].

A good workflow inside the medical team was developed for the management of chronic patients. Pharmacists also had to detect specific severe symptoms (e.g., blue lips or face, dyspnea, chest pain, etc.) [74]. At the same time, the pharmacists had to identify the patients who traveled in risk areas [13]. The COVID-19 patient counseling could be undertaken in different ways (video, radio, posters, flyers, etc.): clinical manifestation of the disease compared to flu or cough, hygiene rules, the need of the lockdown, avoiding scares, and stress caused by the disease, reducing self-medication, etc. [81]. Al-Quteimat et al. described the preventive methods in order to reduce the touch of the facial T-zone (nose, mouth, and eyes) [13]. 

Regarding the medication, Novak et al. showed that one-third of pharmacists included in their study responded to patients’ questions about the potential risks of certain drugs (e.g., angiotensin-converting-enzyme inhibitors—ACEIs, angiotensin II receptor blockers—ARBs, nonsteroidal anti-inflammatory drugs—NSAIDs, glucocorticoids), and more than 70% of them about some drugs with potential curative or preventive properties in COVID-19 (e.g., certain antibiotics, antivirals, chloroquine) [23]. Despite the importance of this information, in many situations, the pharmacists could not completely advise patients in the pharmacy. The Croatian and Serbian pharmacists identified that the difficulties in communication were determined by queues or window drug dispensation. It was encouraging that over one-fifth of the questioned pharmacists provided online counseling [23]. Additionally, in Croatia, pharmacists provided written information especially to the elderly [42]. Moreover, pharmacists could organize workshops for the community (schools, community centers, etc.) [13] or could provide educational flyers to patients [45]. Okoro noticed that the pharmacist’s role is important in patient counseling for reducing unjustified purchases or to correctly use ACEIs or ARBs. All pharmacist’s interventions led to increased adherence and the reduction in anxiety and stress [82]. Nonetheless, the pharmacists educated people regarding the increased risks of pathogenic infection after eating raw meat or animal products, or visits to animal markets [14]. 

### 6.3. Online Purchasing and Home Drug Delivery

After WHO declared the pandemic, the internet represented a main source of obtaining information about drugs or pathologies or purchasing drugs, PPE, disinfectants, including self-diagnosis. These practices can be very dangerous for patients’ health. In this context, the practice of online pharmacies increased a lot. For example, a study assessed the online drug-purchase activity and the motivational factors for the online buying decision. The results showed that over one-third of the respondents purchased drugs from the internet, most of them being single older men. The most demanded products were dietary supplements, analgesics, antihistamines, or anti-cough drugs [83].

On the other hand, the pandemic intensified the activity of home-delivery for drugs PPE and disinfectants. This service focused on patients with COVID-19, the elderly, and patients with chronic diseases. In some countries (e.g., Germany, Finland, Latvia, UK), this service (drug home delivery and collect boxes) is remunerated [35]. The Australian Government introduced a new pharmaceutical service for home delivery of drugs and the Repatriation Pharmaceutical Benefits Scheme drugs. This service was subsidized for quarantined or vulnerable people [38]. The pharmacies could choose personal delivery (e.g., Portugal, UK), courier delivery (e.g., in Portugal based on the agreement between Associação Nacional das Farmácias and the Portuguese post office) or delivery by the Red Cross (e.g., Croatia, Italy, Spain). Thus, the Red Cross collaborated with local community pharmacies to ensure home delivery of medicines [48,84]. Over 22% of Croatian pharmacists and over one-third of Serbian pharmacists delivered drugs to patients’ homes [23]. The patients with chronic kidney disease were a special category because they need specific medications and dialysis consumables while being a category with a very high COVID-19 risk. Okoro presented the involvement of community pharmacists in the home delivery of these products for patients with chronic kidney disease [82]. 

### 6.4. COVID-19 Testing

In many countries, pharmacists have experience in conducting point-of-care tests for glycaemia, cholesterol, blood pressure or streptococcus A antigen, pharmacogenetic profile, etc. Hess et al. noticed that the involvement of pharmacies in COVID-19 testing played a vital public health role in mitigating the transmission of this infection [74]. On this basis, the authorities from 15 European countries (Austria, Belgium, the Czech Republic, France, Germany, Ireland, Italy, Malta, the Netherlands, Portugal, Romania, Spain, Sweden, Turkey, UK) involved pharmacists in COVID-19 testing, either by PCR (Polymerase chain reaction) or by rapid tests [22,35]. Moreover, in Germany pharmacists could perform rapid antigen tests for a fee and provide free FFP2 masks [60].

### 6.5. Vaccine Administration

In some states (e.g., UK, USA, etc.) pharmacies provide flu vaccination services. Even before the approval of the first COVID-19 vaccine, Lee et al. noted the importance of the pharmacies and pharmacists in COVID-19 vaccination. Thus, in order to ensure the success of this process, the authorities should include pharmacies in vaccine administration [85]. Hess et al. considered that the inclusion of pharmacists as vaccinators increased the number of doses administered per week (up to 25 million), which would contribute to a faster immunization of the population [74]. At the moment, many countries (Australia, Belgium, France, Ireland, Italy, Norway, Poland, Portugal, UK, USA) permitted the administration of the COVID-19 vaccines by pharmacists (including trained pharmacy students in France) [35,85]. In the USA, the trained pharmacists and technicians are considered vaccine service providers by the CDC [86]. Despite all these positive aspects, not all pharmacists wanted to be involved in vaccine administration in the pharmacy [87].

### 6.6. Combating the Toxic “Infodemic”

After the pandemic outbreak, the abundance of information equally came from academic and non-academic media. Between 1 January 2020 and 22 April 2020, over 5000 scientific papers on the COVID-19 issue were published [88]. Easy Internet access was an important factor in getting information from anywhere, and social media had proven to influence the attitudes, convictions, and behaviors of the entire population. Since the beginning of the pandemic, the WHO General Director Tedros Adhanom Ghebreyesus stated that the dissemination of misinformation led to the “Infodemic” [89]. For example, in a study carried out by Kouzy et al. in the first weeks of the pandemic it was observed that over 40% of Twitter posts included misinformation or unverifiable information [90]. The same conclusion was reported in Italian media by a study conducted by Rovetta et al. between January and March 2020 [91]. 

To combat the Infodemic, the pharmacist could verify the sources through scientific methods, could recognize the misinformation, and could adapt their language to the understanding capacity of every person [92]. Also, they could find out the patients’ information searching behavior (what and where they search). On the other hand, they could recommend patients to the official websites of the authorities or of the public health organizations [14].

## 7. Specific Clinical and Hospital Pharmacy Services

### 7.1. Clinical Pharmacists’ Roles

Some studies present the clinical pharmacists’ roles during this period, such as: (a) therapeutic issues (identifying, prevention, treating); (b) therapeutic alternatives for out-of-stock drugs; (c) patients’ counseling including medication review (especially by phone, or by video conferences); (d) providing information about new therapies and about the safety, interactions or ADRs of the drugs (especially ibuprofen, glucocorticoids, ACEIs, BRAs, etc.); (e) contribution in clinical study teams; (g) elaboration of clinical guides together with doctors (dosage, precautions, interactions, ADRs, contraindication, special risk categories, etc.); (h) nurses and paramedic staff education on medication issue, reporting of ADRs, PPE usage, or manipulating the samples and residual materials from patients; (i) prevention of the COVID-19-related stigma through combating the negative behaviors or language and providing psychological counseling; (j) antimicrobial stewardship (collaboration with microbiology laboratories for assessment of COVID-19 tests, monitoring the patients’ compliance, drafting and implementing for the use of the antivirals or other drugs, management of the shortages, and the investigation of new drugs for the infection treatment) [12,13,42,81,93,94,95,96,97,98,99,100]. For example, Serbian pharmacists searched and evaluated the information about remdesivir [42]. Wang et al. showed that in a Chinese hospital with 2500 beds, 96% of the recommendations provided by the clinical pharmacist were accepted (e.g., discontinuation of treatment, choosing other medication, introducing a new drug in the therapeutic plan, dose adjustment, monitoring suspected ADRs or drug concentration, etc.). These are related to ADRs or treatment with antibiotics, antivirals, glucocorticoids, antifungal agents, mechanic ventilation, renal replacement therapy, artificial liver support [97]. Li et al. noticed the pharmacists’ assessment of the risk of stress-induced gastric mucosa damage, and the risk of venous thromboembolism [101]. The pharmacists from different institutions offered logistic and clinical support to obtain drugs for off-label use (e.g., remdesivir) through patient eligibility assessment, preparation of the file, and obtaining the authorization from the manufacturer and authorities to use these drugs [102].

During the pandemic, the activity of clinical and hospital pharmacists was completely disturbed. McConchie et al. reported a significant reduction in patient numbers and an increased number of patients in the intensive care unit. The perturbations led to the decreasing of drugs orders and prescriptions and even the relocation of the clinical pharmacist from the operatory room in the pharmacy. However, medication reviews increased in number, as well as the quantity of intravenous compounding drugs prepared, the focus being on specific drugs used in COVID-19 (fentanyl, midazolam, azithromycin, hydroxychloroquine, enoxaparine, propofol, neuromuscular blockers, etc.). Regarding clinical pharmacy activity, the same study reported: (a) the interventions of the pharmacists for drug pharmacokinetic monitoring (e.g., warfarin, vancomycin, aminoglicosides, anticoagulants), (b) patient phone counseling, (c) medication review (e.g., antibiotics; dose adjustment, i.v.-oral conversion, reorganization of the therapeutic plan for reducing the number of visits to patients, monitoring the drugs’ ADRs, especially of hydroxychloroquine and azithromycin, etc.); (d) offering medical solutions; (e) interdisciplinary collaborations, etc. [103]. According to another study, pharmacists were responsible for evaluating and summarizing the scientific literature, guidelines, or health organizations’ recommendations, disseminating this information, presenting the updated inventory, etc. [61].

### 7.2. Collaboration with the Medical Team

The presence of pharmacists in healthcare teams is essential during a crisis, especially during the outbreak [29,61]. Since the beginning of the pandemic, pharmacists elaborated emergency drugs formularies, therapeutic plans, or documents with information on drugs used in COVID-19 in order to support the doctors’ activity; they participated in medical teams for treating COVID-19 patients with comorbidities. In addition, alongside doctors, they assessed the treatment. Another important part of their practice was to monitor drug ADRs and drug interactions, especially in critical patients or patients treated with narrow therapeutic index drugs [31,76,97,101,104]. Clinical pharmacists had to ensure that the off-label drug usage was appropriate. Thus, they had to monitor the treatment that could cause ADRs and adjust the doses depending on organ damage. The pharmacological meetings with the medical team were performed by alternative communication methods such as videoconferences or WhatsApp and all therapeutic adjustments were made with the involvement of pharmacists [104].

Among the collaborations of the clinical pharmacist with the medical teams for the treatment of COVID-19 patients, we can mention the proposal of the clinical pharmacist from the intensive care unit of the University Hospital of Amiens (France) for pulmonary administration of interferon-β-1b, after nebulization. Mary et al. showed that the 4 patients who did not respond to any other treatment had a favorable evolution after interferon administration [105].

According to the results of a study published by Severino et al., the active role of the pharmacist in the medical team was to advise on the type of artificial nutrition used and its monitoring, malnutrition being common in reduced-mobility intensive care patients. Inflammatory processes and COVID-19 sepsis also directly contributed to malnutrition. Moreover, the pharmacist’s intervention is very important for patients with post-intubation dysphagia for adding the medication in the enteral nutrition following some procedures (e.g., splitting tablets, chopping, crushing or spraying tablets, opening capsules, mixing and diluting liquid forms, etc.). Another presented role was in-home nutrition programs and patient education [106].

A prospective study conducted in Lille University Hospital (France) showed that there was no difference in pharmacist’s interventions between a COVID-19 patient group and non-COVID-19 group (e.g., addition or dosage modifications of anticoagulant agents, combating over-dosage or over-prescription of proton pump inhibitors, avoiding combinations of bronchodilators from the same class, monitoring of prescriptions with broad-spectrum empirical anti-infective agents, etc.) [107]. 

A study conducted in 16 EU countries reported that pharmacists complained about limited recognition of their competence by the medical team or by the organizational management. The same study highlighted the lower acceptance rate of pharmacists’ interventions by the Swiss. Another problem noticed in Denmark referred to divergent information about experimental drugs received by the medical team staff at the beginning of the pandemic [42]. 

### 7.3. Remote Patient Interaction

For pharmacists, the pandemic period means the manifestation of their creativity. Thus, they had to use different ways of distance communication with patients or medical teams, like telephone, WhatsApp, FaceTime or Skype, written interaction including website-based or Internet platforms (e.g., Pharmadoctor eTool in the UK), video conferences, or even television and radio. All information was supposed to reduce anxiety and increase treatment adherence [48,56,76,93,101]. The telepharmacy concept was implemented a few years ago, and it represents the delivery of hospital pharmacy services. During the COVID-19 pandemic, the access to the Clinical Pharmacist helpline service from an Inflammatory Bowel Disease unit increased by 228% compared to the pre-COVID-19 pandemic period [108]. A study conducted in Spain showed that several Spanish hospitals introduced online counseling and home drug-delivery services (external courier or hospital’s own transport services) forced by the Coronavirus crisis. Thus, a higher number of patients benefited from telepharmacy services. Even if the communication was online, it abided by all the face-to-face procedures, and all pharmacists’ interventions were mentioned in the patients’ electronic medical file [26]. A study from a cardio-oncology clinic showed that the clinical pharmacists assisted the medical team in a virtual hybrid approach with discussions and medication prescriptions (including review, education, drug interactions, or titrations) in cardiovascular diseases and smoking cessation [109]. However, the limited access to the internet for patients or even for pharmacists was reported, which made communication difficult [42].

### 7.4. Creative Solutions in Hospitals

Regardless of where the pharmaceutical care took place, the pharmacists disseminated information about the COVID-19 issue. Temporary ambulatory pharmacies were set up to provide drugs only for patients with fever or for COVID-19 suspected patients. It was also necessary to set up Fangcang hospitals for the hospitalization of patients. Pharmacists that worked in classic hospitals or in Fangcang shelter hospitals were skillful to provide accurate, complete, and concise information adapted to the level of understanding of each patient. The information was disseminated by different media (TV, Twitter, Internet, Tik Tok, Bilibili, Youku, YouTube, radio from Fangcang hospitals, online remote pharmacies, etc.) [31,94,100,101]. Moreover, Goff et al., noticed that a pharmacist from the Ohio State University Wexner Medical Center in Columbus had over 20 interviews with local television health reporters, national news media, or different magazines in order to provide COVID-19 education to the public [94]. 

Using a mobile phone application, pharmacists monitored the anticoagulant therapy and optimized the warfarin doses for 500 patients [76]. Another creative solution was presented by Elbedini et al. in order to deal with the pressurized metered-dose inhalers with salbutamol shortage, thus reusing the incomplete used inhalers to treat multiple patients [44,110]. 

### 7.5. Limiting Drug Shortages

In hospitals, the pharmacists estimated the maximum consumption of essential medicines for the total number of COVID-19 patients. Based on these estimations, the inventory was updated which allowed earlier identification of new or worsening shortages [41]. Since the beginning of the state of emergency, the Spanish healthcare authorities regulated the drugs dispensed in hospitals [26]. Before the pandemic, in a Pakistani hospital, over 15% of the dispensed drugs remained unused. To limit this waste of drugs, the pharmacy dispensed drugs only for single-use, because it was forbidden to return them to the pharmacy from the COVID-19 wards [104].

**Figure 2:**
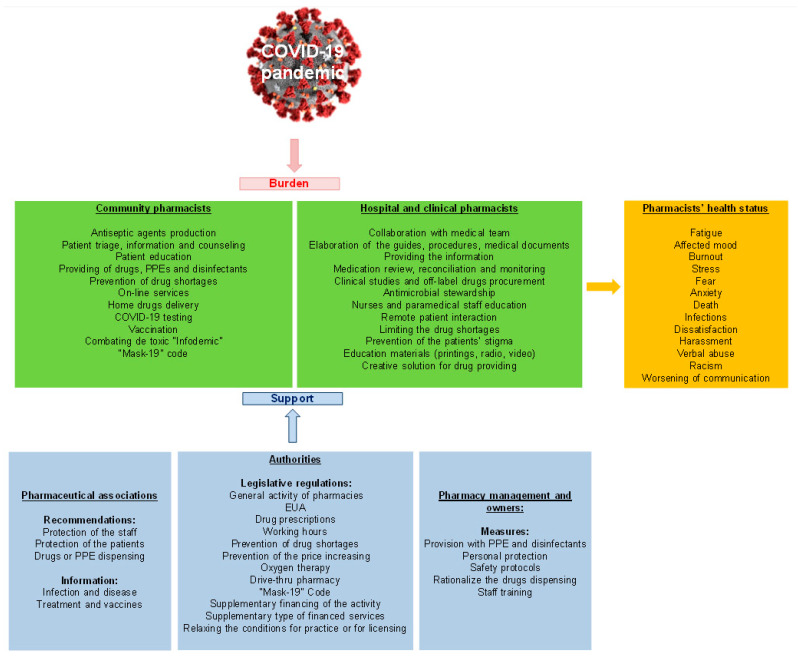
Pharmaceutical care: practice and influencing factors. PPE—personal protective equipment, EUA—emergency use authorization.

The aspects previously discussed were summarized in Figure 2. Thus, the burden of COVID-19 on pharmaceutical care, the influences of the authorities, professional organizations, and managers and owners on this issue were addressed. Sometimes, because of all the pressure, the pharmacists’ mental and health status could have been affected.

Since the beginning of the COVID-19 crisis, pharmacists were involved in different activities that lead to better management of the pandemic. They had a complex role in health product dispensing, patients’ counseling, medical teams, community-care, and as supporters for authorities’ activities (Table 1). So, the pharmaceutical services were diverse and essential for protecting the health status of the population, particularly to improve the life quality of COVID-19 patients. During this period, pharmacists achieved new skills and played an important role in healthcare and in crisis management. 

## 8. Limitations of the Study

This study presents several aspects regarding pharmaceutical care in the COVID-19 pandemic, but it has its limitations. The published studies are heterogeneous because the pharmaceutical services are not standardized worldwide. In this respect, our paper tried to offer an overview of pharmaceutical care during the COVID-19 pandemic, even if the level of identified evidence in scientific literature was not particularly complex. Also, the information is not structured on geographical areas because of the lack of homogeneity of the data identified in different publications or public data. The region-structured information could be useful to compare the authorities’ decisions and the impact of the COVID-19 pathology on the pharmaceutical care practice.

## 9. Conclusions

The pandemic offered the opportunity to develop the pharmaceutical care practice, and to prove the pharmacists’ skills, involvement, professionalism, and creativity to the authorities and the entire medical community. The pharmacist is an academic leader and a medical advice provider for patients, medical teams, as well as for the entire population. Beyond the risks, pharmacists have adapted their pharmaceutical care practices, extending their services, and being motivated by a strong sense of professionalism and humanity. Throughout their practice, they added value to the healthcare system in the fight against COVID-19. Together with this crisis, pharmaceutical care entered a new phase, demonstrating the ability of pharmacists to be competent and accessible providers of public health. The pharmaceutical care services are not standardized, differing from country to country, and dependent on the capacity and desire of each national authority to apply them as the main part of public health. It remains to be seen how much these services will evolve and what the official recognition of pharmacists’ activities by each authority or institution will be. Future studies should present a way to standardize pharmaceutical care activity. These could provide authorities a tool to monitor and even fund these services, which would help the healthcare system and improve the patients’ quality of life. Such tools would also be useful to react to a future health crisis.

## Figures and Tables

**Table 1:** A brief overview of pharmaceutical care during the COVID-19 pandemic.

Pharmacists’ Role	Pharmaceutical Services
I. Management of health product dispensing	Adequate management of drugs, dietary supplements, PPE or sanitizers supplying
	Stock management efficiency
	Limitation of the released quantity of the most requested products
	New tasks for pharmacists: delivery of medical oxygen, renewal of prescriptions for chronic diseases, drugs substitution for chronic treatment (including hypnotics, anxiolitics, narcotics or opioid drugs) etc.
	Offering of therapeutic alternatives
	Manufacturing of antiseptic agents in order to avoid shortages
	On-line services (e.g., electronic prescriptions)
	Home-delivery
	Drive-thru services
II. Interaction with patients	COVID-19 patients’ triage (including the differentiation from flu and cold)
	Detecting specific severe symptoms of COVID-19
	Counselling regarding hygienic rules and the importance of isolation and lock-down
	Medication review
	Counselling patients reffering to infection control and treatment (e.g., disease, symptoms, medication, ADRs etc.)
	Reducing self-medication
	Providing written information
	Preventing and combating the COVID-19 related stigma, scares, stress etc.
III. Medical team	Elaborating the COVID-19 specific documents, therapeutic plans, recommendations or guides
	Colaboration with medical team (e.g., new drugs, initiating of a new treatment, patient follow-up, clinical studies, antimicrobial stewardship etc.)
	Medication review and reconciliation
	ADRs, interactions or drug pharmacokinetic monitoring
	Staff training regarding PPE usage, reporting of ADRs, manipulation of biological samples or residual materials from patients
IV. Community-care	Providing information regarding hygiene rules, importance of look-down or isolation
	Counselling for reducing self-medication or unjustified buying of health products
	Elaboration of educational materials: written (e.g., flyers, posters, brochures etc.) and audio-video materials broadcasted on TV, radio or social-media
	Counselling in order to avoid COVID-19 related stress, scare or stima
	Organization and participation to workshops on COVID-19 topics
V. Support for authorities	Combating the misinformation or unverifiable information
	Logistic and clinical support to obtain drugs for off-label use in COVID-19 disease
	COVID-19 testing
	Vaccine administration (including COVID-19 vaccines)
	Involvement in “Mask-19” project
	Providing free PPEs equipment through Government projects

PPE—personal protective equipment, ADR—adverse reaction.
